# Regulation of Mitochondrial Homeostasis and Metabolic Programming in Memory B cells by Mitophagy

**DOI:** 10.1080/27694127.2022.2061681

**Published:** 2022-04-20

**Authors:** Marietta M. Budai, Min Li, Srikanth Kodali, Min Chen, Jin Wang

**Affiliations:** a Immunobiology and Transplant Science Center, Houston Methodist Research Institute, Houston, TX, USA; b Department of Pathology and Immunology, Baylor College of Medicine, Houston, TX, USA; c Department of Surgery, Weill Cornell Medical College, Cornell University, New York, NY, USA

## Abstract

The formation of long-lived immune memory cells specific for pathogens is critical for the establishment of long-term immune protection against future infections. BNIP3L/NIX and BNIP3, two functionally redundant BCL2 family members required for mitophagy, undergo significant upregulation after memory B cells are formed. Deletion of *Bnip3l* and *Bnip3* leads to mitochondrial accumulation, and increases in oxidative phosphorylation and fatty acid synthesis, resulting in the loss of memory B cells. These observations suggest that after the formation of memory B cells, mitophagy is critical for clearing superfluous mitochondria to re-shape the metabolic programs, thereby protecting the metabolic quiescence and longevity of memory B cells .

## Protection of immune memory cells by macroautophagy/autophagy

**Figure 1.**
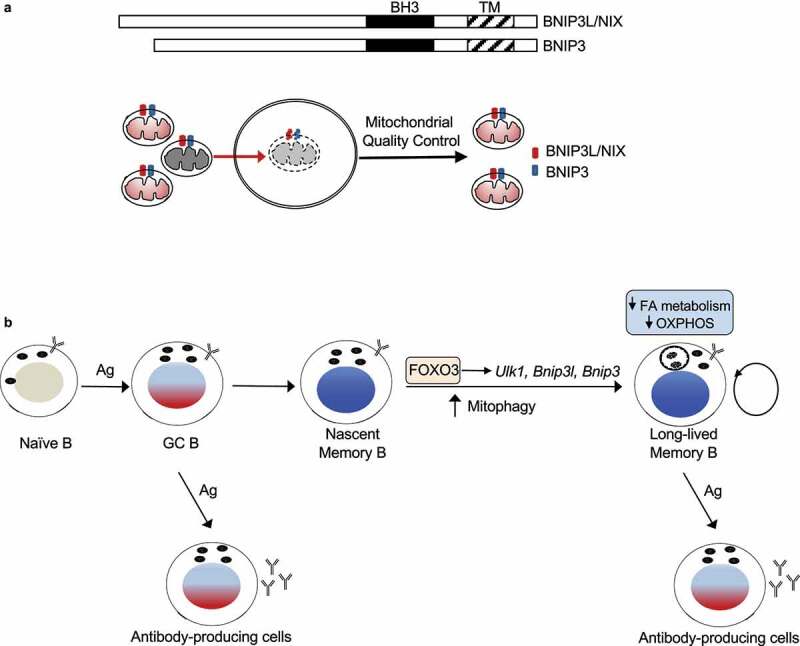
Metabolic programming by mitophagy after the formation of memory B cells. (**A**) BNIP3L/NIX and BNIP3 are two functionally redundant BCL2-family proteins localized on mitochondrial outer membranes. BNIP3L and BNIP3 mediate the targeting of mitochondria for sequestration by phagophores for degradation. BH3, BCL2 homology 3; TM, transmembrane. (**B**) During an immune response, naïve B cells are activated and develop into germinal center (GC) B cells. GC B cells can form memory B cells or antibody-producing cells. Upregulation of FOXO3 after memory B cell formation drives the expression of autophagy genes, such as *Ulk1* and *Atg14*, as well as mitophagy regulators *Bnip3l* and *Bnip3*, to induce mitophagy. Mitophagy is critical for the control of mitochondrial content and the fine-tuning of mitochondrion-dependent metabolic programs, contributing to the formation of metabolically quiescent and long-lived memory B cells. Ag, antigen.

We have previously observed that autophagy is essential for the long-term maintenance but not initial formation of memory B cells. *atg7^–/–^* memory B cells can be partially rescued by ROS scavengers, indicating that oxidative stress-induced cell death is involved in the loss of these cells. Similarly, mice with B cell-specific deletion of *Bnip3l* and *Bnip3* (*bnip3l^−⁄−^bnip3^−⁄−^*) show normal initial formation of memory B cells, but these cells are lost a few weeks later [1]. Although *bnip3l*^−⁄−^ *bnip3*^−⁄−^ memory B cells display increases in mitochondrial content with elevated production of ROS, inhibition of ROS with N-acetylcysteine/NAC does not protect these cells *in vivo*, indicating that inhibition of oxidative stress is not sufficient to protect *bnip3l*^−⁄−^ *bnip3*^−⁄−^ memory B cells. It has been shown that BNIP3L- and BNIP3-regulated mitophagy involves both canonical autophagy and ATG7-independent non-canonical autophagy. Therefore, additional mechanisms for BNIP3L- and BNIP3-dependent mitophagy likely exist for the protection of memory B cells (Figure 1).

## Maintenance of mitochondrial homeostasis by BNIP3L- and BNIP3-dependent mitophagy

B cells activated by antigens and T helper cells undergo rapid proliferation in the germinal center (GC). Proliferating GC B cells require large numbers of mitochondria to generate ATP in order to meet their energy needs. When GC B cells develop into memory B cells, BNIP3L and BNIP3 are upregulated (Figure 1). Deficiencies in BNIP3L and BNIP3 lead to mitochondrial accumulation in memory B cells as shown by increased mitochondrial DNA (mtDNA) and mitochondrial protein staining [1]. These findings suggest that BNIP3L and BNIP3 are required for removing superfluous mitochondria to maintain mitochondrial homeostasis in memory B cells (Figure 1).

## *Dysregulated mitochondrial metabolism in* bnip3l^−⁄−^ bnip3^−⁄−^ *memory B cells*

**Figure 2.**
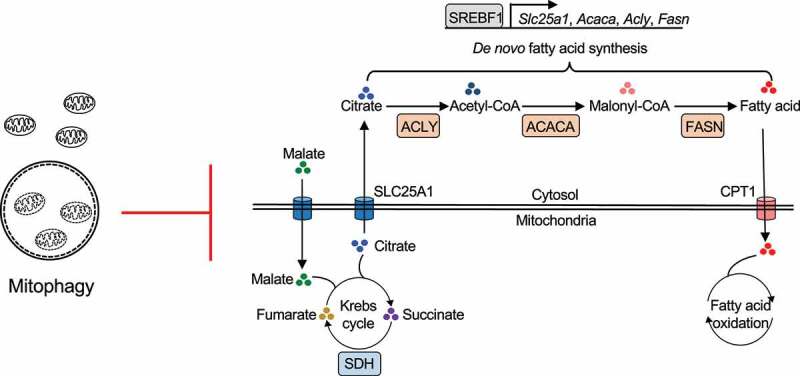
Control of metabolic programs in memory B cells by mitophagy. Citrate from the Krebs cycle is imported by SLC25A1 into mitochondria, to fuel *de novo* fatty acid synthesis through ACLY, ACACA/ACC1 and FASN. Mitophagy plays an important role in controlling mitochondrial content, resulting in decreased OXPHOS and *de novo* FA synthesis.

*bnip3l*^−⁄−^ *bnip3*^−⁄−^ memory B cells display increased levels of oxidative phosphorylation (OXPHOS). Moreover, RNA-seq analyses show that oxidative phosphorylation genes, *Sdhc* and *Sdhaf2* of the succinate dehydrogenase/SDH complex (Figure 2), are elevated in *bnip3l*^–/–^ *bnip3*^−⁄−^ memory B cells, consistent with the increased OXPHOS capacity in these cells. Interestingly, *de novo* fatty acid (FA) synthesis-related genes, including *Acly* (ATP citrate lyase), *Acss2*, and *Acsf3*, are also among those genes increased in *bnip3l*^–/–^ *bnip3*^−⁄−^ memory B cells [1]. It has been shown that active mitochondria can stimulate *de novo* fatty acid synthesis. The mitochondrial citrate transporter (SLC25A1) can transport citrate from the Krebs cycle to the cytosol for *de novo* FA synthesis catalyzed by ACLY, ACACA/ACC1 (acetyl-coA carboxylase alpha), and FASN (fatty acid synthase) (Figure 2). We find that *de novo* FA synthesis genes and their master regulator, SREBF1 (Figure 2), are upregulated in *bnip3l*^–/–^ *bnip3*^−⁄−^ memory B cells. *bnip3l*^–/–^ *bnip3*^−⁄−^ memory B cells accumulate lipid droplets as measured by LipidTOX staining, whereas blocking mitochondrial citrate export with a highly specific SLC25A1 inhibitor, CTPI-2, inhibits lipid accumulation and rescues *bnip3l*^–/–^ *bnip3*^−⁄−^ memory B cells [1]. These findings indicate that the accumulation of active mitochondria in the *bnip3l*^–/–^ *bnip3*^−⁄−^ memory B cells leads to the enhanced export of citrate via SLC25A1 to fuel excessive *de novo* FA synthesis, resulting in lipid accumulation and the loss of memory B cells.

## *Mechanisms for the loss of* bnip3l^–/–^ bnip3^−⁄−^ *memory B cells*

We detected the active phosphorylated form of a necroptotic signaling molecule, RIPK3, in memory B cells [1]. Moreover, silencing of RIPK3 rescues *bnip3l*^–/–^ *bnip3*^−⁄−^ memory B cells measured by adoptive transfer experiments *in vivo*, suggesting that the loss of *bnip3l*^–/–^ *bnip3*^−⁄−^ memory B cells involves necroptosis [1]. During necroptosis, activated RIPK1 phosphorylates RIPK3 to form the RIPK1-RIPK3 necrosome, which in turn phosphorylates MLKL and recruits it to the membrane as a pore-forming protein to induce necroptosis. Excessive fatty acids can covalently bind to MLKL by a modification called fatty acylation, which promotes necroptosis. It will be important to determine whether lipid droplet accumulation in *bnip3l*^–/–^ *bnip3*^–/–^ memory B cells induces fatty acylation of necroptotic molecules to trigger necroptosis.

Memory B cells maintain their long-term persistence through both self-renewal and inhibition of cell death. In addition to increased cell death, reduced self-renewal potentials can contribute to the loss of *bnip3l*^–/–^ *bnip3*^–/–^ memory B cells. The PDCD1LG2/PD-L2^–^ CD80^–^ subset of memory B cells displays self-renewal capacities. PDCD1LG2^–^ CD80^–^ memory B cells show certain stem cell properties, with higher expression of cell cycle genes and the capacity to form new germinal centers. Emerging evidence suggests that mitophagy plays an important role in the maintenance of stemness and self-renewal in progenitor cells. It will be important to determine whether mitochondrial accumulation in *bnip3l*^–/–^ *bnip3*^–/–^ memory B cells causes dysregulated metabolism and decreased stemness, leading to reduced self-renewal.

Our study reveals a critical role for BNIP3L- and BNIP3-mediated mitophagy in the regulation of mitochondrial homeostasis, to establish metabolic quiescence and protect the longevity of memory B cells (Figure 1). Several questions still remain to be addressed. What is the molecular mechanism for BNIP3L and BNIP3 to mediate mitophagy in memory B cells? How do the cells sense the superfluous mitochondria and target them for clearance by mitophagy? Do both decreases in the self-renewal potentials and increases in cell death contribute to the loss of *bnip3l*^–/–^ *bnip3*^–/–^ memory B cells? Answering these questions will advance our understanding of how mitophagy regulates metabolic re-programming to support the persistence of memory B cells, and to maintain the long-term humoral immune memory. Our studies may also facilitate the design of more effective vaccines against pathogens.
